# Reduced plasma level of diazepam-binding inhibitor (DBI) in patients with morbid obesity

**DOI:** 10.1007/s12020-014-0522-5

**Published:** 2015-01-06

**Authors:** Agnieszka Siejka, Joanna Jankiewicz-Wika, Henryk Stępień, Jolanta Fryczak, Jacek Świętosławski, Jan Komorowski

**Affiliations:** 1 Department of Clinical Endocrinology, Medical University of Lodz, ul. Sterlinga 3, 91-425 Lodz, Poland; 2 Department of Immunoendocrinology, Medical University of Lodz, ul. Sterlinga 3, 91-425, Lodz, Poland; 3 Department of Neuroendocrinology, Medical University of Lodz, ul. Sterlinga 3, 91-425 Lodz, Poland

## Introduction

Peptides of the endozepine family, including diazepam-binding inhibitor (DBI), are regulatory neuropeptides originally isolated from rat brain tissue as factors potentially able to displace benzodiazepines from their binding sites [1]. DBI is widely expressed in the central nervous system, and high concentrations have been found in areas involved in the control of feeding behavior [1]. The biological effects of endozepines are mediated through three types of receptors: central-type benzodiazepine receptors (CBR), peripheral-type benzodiazepine receptors (PBR), and metabotropic receptors [1, 2]. It has been shown that injection of octadecaneuropeptide (ODN), which is a derivative of DBI, causes reduction in food consumption. Its anorexigenic effect is long-lasting, and leads to a substantial loss of weight [3].

Obesity is a worldwide epidemic leading to multiple complications. The term ‘morbid obesity’, or class III obesity according to the WHO, is used to describe adults with a body mass index (BMI) of 40 kg/m^2^ or more with significant medical problems caused by their weight. As there is currently lack of effective pharmacological therapy, efforts are underway to identify new factors which may be involved in its pathophysiology [4], and which possibly may be a target for future treatment or prevention.

Pathophysiology of obesity is complex and not completely understood. Nevertheless, studies on the neuromodulatory changes in obese subjects [5–7], together with recent observations of endozepine pharmacology [1, 2], suggest that DBI, or one of its derivatives, may be a potential candidate for further research. This is the first pilot study to investigate, and confirm, the reduced plasma peripheral blood DBI concentration in patients with morbid obesity.

## Materials and methods

### Patients

The test subjects comprised 58 patients (26 men, 32 women), mean age 43.43 ± 11.08 years, with morbid obesity (BMI 48.17 ± 7.52 kg/m^2^) and 19 normal-weight controls (BMI 21.59 ± 2.5 kg/m^2^; 5 men, 14 women; mean age 35.5 ± 15.37 years). The morbid obese subjects have been further subdivided into: patients with diabetes mellitus (DM+), patients with impaired fasting glucose and/or impaired glucose intolerance (Prediabetes+) and subjects with normal glucose tolerance (NGT). The glucose status of three of the patients could not be assessed based on the data. The characteristic of the subgroups is presented below. DM+ group comprised 18 patients (7 men, 11 women), mean age 51.27 ± 2.287 years, BMI 47.576 ± 1.329. Prediabetes group comprised 20 patients (13 men, 7 females), age 41.04 ± 2.16, BMI 50.39 ± 1.828. Morbid obese group with NGT comprised 17 patients (6 men, 11 females), mean age 38 ± 2.39, BMI 45.471 ± 1.63. All patients with diabetes mellitus used at least one of the antidiabetics (9 patients used metformin only, 5 patients used combination of metformin and sulfonylurea, 3 used combination of metformin and insulin, and 1 insulin only). The study has been approved by the Local Ethical Committee of the Medical University of Lodz and conducted in accordance with the Helsinki Declaration.

### Methods

Peripheral blood samples were drawn in the morning after overnight fasting. Plasma leptin (Ob; Labor Diagnostica Nord GmbH&Co. KG, Germany, sensitivity—0.5 ng/ml; inter-assay precision—5.0 %); soluble leptin receptor (sOb-R; BioVendor; EU; sensitivity—0.04 ng/ml; inter-assay precision—7.23 %), cholecystokinin (Phoenix Pharmaceuticals, Inc., cat. No. EKE-069-04, range 0–100 ng/ml) and DBI) (Sunred Biotechnology, sensitivity—0.688 ng/ml; inter-assay precision—<12 %). Blood glucose was also analyzed. Results of other biochemical parameters, such as glucose and insulin, were measured during in-patient stays. Furthermore, BMI was calculated. The homeostasis model assessment insulin resistance index [HOMA-IR = fasting insulin (mIU/L) × fasting glucose (mmol/L)/22.5] was calculated. The AUC was calculated using an average of a number of trapezoidal approximations.

### Statistics

All comparisons were made using Statistica 10 software. The one-way Anova followed by the Tukey test was used, as well as the Student’s paired *t* test. The relationship between features was evaluated by Pearson’s linear correlation coefficient analysis. The values are presented as the mean ± SEM. A *p* value <0.05 was considered statistically significant.

## Results and discussion

DBI and its peptide fragments, including ODN, which bind benzodiazepine receptors, are known as endozepines. They are specifically produced by various cells, mainly hypothalamic astrocytes and tanycytes [8–10]. These peptides are involved in the regulation of food intake, and ODN reduces body weight in rodents [3]. Moreover, endozepines seem to play a critical role in brain glucose sensing and are potentially new targets in the treatment of metabolic disorders [2].

**Fig. 1:**
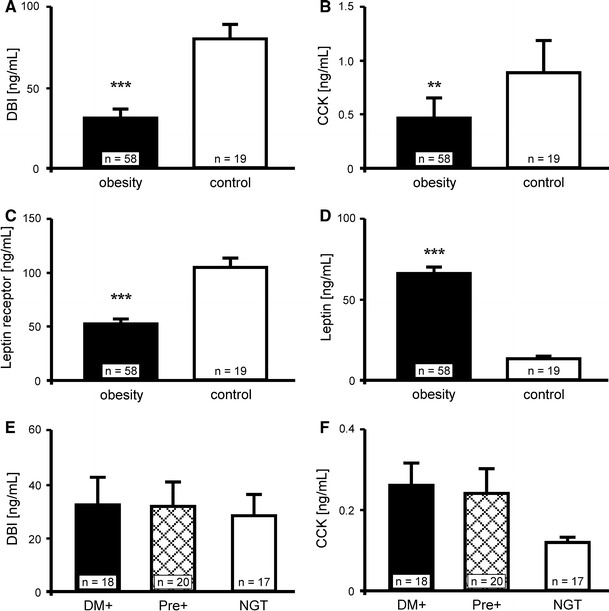
Blood levels of **a** diazepam-binding inhibitor (DBI), **b** cholecystokinin (CCK), **c** soluble leptin receptor (sOb-R) and **d** leptin in obese subjects, and in controls. The graphs represent mean ± SEM. **p < 0.01 versus control; ***p < 0.005 versus control; *n* number of subjects. DBI and CCK correlation to glucose tolerance status in morbidly obese patients (**e** and **f**, respectively). DM—morbid obese patients with diabetes mellitus; Pre+—morbid obese patients with prediabetes (defined as impaired fasting glucose or/and impaired glucose intolerance); NGT morbid obese patients with normal glucose tolerance. The graphs represent mean ± SEM. *p < 0.05 versus NGT. *n* number of subjects

The discovery that adolescents with anorexia nervosa demonstrate reduced fasting plasma levels of DBI [11] prompted this study of DBI levels in obese subjects. Surprisingly, in a similar way to the depressed DBI levels demonstrated in anorexia nervosa, the levels of DBI in obese patients were also found to be significantly lower, as compared to normal-weight subjects (31.11 ± 5.06 vs 80.98 ± 8.33 ng/ml, respectively; Fig. 1a). There was no statistical difference in the concentrations of DBI between the female and male patients (31.77 ± 6.68 vs 30.27 ± 7.88 ng/ml, *p* > 0.05). In order to exclude false results, the levels of leptin and leptin receptor (sOb-R) were measured in the same blood samples. As expected [4], leptin levels were found to be significantly higher and sOb-R significantly lowered in obese patients, as compared to controls (Fig. 1c, d). Leptin and cholecystokinin (CCK-8) inhibit food intake [12], but effect of leptin is lowered by high concentrations of sOb-R.

It has been previously shown that DBI, a trypsin-sensitive cholecystokinin-releasing peptide (CCK-RP), is secreted from the proximal small bowel, and mediates the feedback regulation of pancreatic secretion and postprandial release of CCK [13, 14]. Significantly reduced concentrations of CCK were identified in the patients with morbid obesity in the present study, as compared to the controls with normal body weight (0.268 ± 0.048 vs 0.58 ± 0.104 ng/ml, respectively; Fig. 1b).

The DBI derivative, ODN is known to possess an anorexigenic activity and this is mediated through activation of the metabotropic receptor [2]. Recent findings have revealed that endozepines play an important role in central glucose sensing. It has been shown that glucose stimulates both endozepine expression in hypothalamic tanycytes, which are directly glucose-sensitive, and endozepine release from hypothalamic explants [2]. It has been further demonstrated that feeding behaviors induced by glucose or d-glucose were reversed by the intracerebroventricular injection of an ODN antagonist or agonist, respectively [2]. In addition, a central injection of ODN antagonist significantly increased blood glucose levels, suggesting that endogenous endozepines tonically reduce glycemia [2]. DBI isolated from the intestine was found to inhibit both early and late phases of glucose-induced insulin release from isolated perfused rat pancreas [15].

In our obese subjects, DBI, leptin, and sOb-R levels did not differ significantly between patients with diabetes and subjects with NGT; BMI did not differ significantly between those groups. Although slightly higher levels of DBI were seen in diabetics, the differences were not statistically significant (diabetics 32.34 ± 9.94 ng/ml vs controls: 28.6 ± 8.6 ng/ml)—Fig. 1e. CCK levels were higher in patients with diabetes (0.26 ± 0.056 ng/ml) vs morbid obese ones with NGT (0.118 ± 0.01 ng/ml), however, there was no significant difference (Fig. 1f). DBI levels did not differ between NGT subjects and patients with prediabetes, defined as impaired fasting glucose and/or impaired glucose intolerance (Fig. 1e). However, leptin was significantly higher (79.17 ± 5.77 vs 56.86 ± 8.64 ng/ml; *p* = 0.03) and sOb-R was significantly lower (39.04 ± 2.4 vs 54.3 ± 7.71 ng/ml; *p* = 0.04) in prediabetic patients (data not shown). Again, CCK was significantly higher in prediabetes than NGT (0.24 ± 0.046 vs 0.118 ± 0.011 ng/ml; *p* = 0.023), as shown in Fig. 1f. CCK correlated positively with fasting insulin level (*R* = 0.296, *p* < 0.05), HOMA value (*R* = 0.345, *p* < 0.01) and BMI (*R* = 0.333, *p* < 0.05).

These findings are the first to demonstrate that DBI levels are significantly reduced in morbidly obese subjects. However, no clear relationship could be identified between this peptide and parts of the glucose-insulin system, although such a correlation could be apparent when larger cohorts are studied. Plasma DBI levels were not directly correlated to body weight in our study (*R* = 0.0862, *p* = 0.52), suggesting that other regulatory mechanisms may be involved in the altered feeding behavior of obesity. Indeed, DBI regulates the production of neurosteroids, which include molecules with opposite orexigenic actions, such as allopregnanolone and dehydroepiandrosterone sulfate (DHEA-S), modulating feeding behavior and body weight positively and negatively, respectively [6, 11]. The precise mechanism by which DBI and other endozepines regulate feeding behavior will need further investigations.
